# Site conditions are more important than abundance for explaining plant invasion impacts on soil nitrogen cycling

**DOI:** 10.1002/ecs2.2454

**Published:** 2018-10

**Authors:** Marissa R. Lee, S. Luke Flory, Richard P. Phillips, Justin P. Wright

**Affiliations:** 1 Department of Biology, Duke University, Durham, North Carolina 27708 USA; 2 Department of Biological Sciences, George Washington University, Washington, D.C 20052 USA; 3 Department of Biology, Indiana University, Bloomington, Indiana 47405 USA; 4 Agronomy Department, University of Florida, Gainesville, Florida 32611 USA

**Keywords:** invasive species, *Microstegium vimineum*, nitrogen, structural equation modeling

## Abstract

Invasive plant species can alter critical ecosystem processes including nitrogen transformations, but it is often difficult to anticipate where in an invaded landscape, these effects will occur. Our predictive ability lags because we lack a framework for understanding the multiple pathways through which environmental conditions mediate invader impacts. Here, we present a framework using structural equation modeling to evaluate the impact of an invasive grass, *Microstegium vimineum* (*M.v*.), on nitrogen cycling based on a series of invaded sites that varied in invader biomass and non-*M.v*. understory biomass, tree basal area, light availability, and soil conditions. Unlike previous studies, we did not find an overall pattern of elevated nitrate concentrations or higher nitrification rates in *M.v*.-invaded areas. We found that reference plot conditions mediated differences in mineralization between paired invaded and reference plots at each site through indirect (via *M.v*. biomass), direct, and interactive pathways; however, the strongest pathways were independent of *M.v*. biomass. For example, sites with low reference soil nitrate and high non-*M.v*. understory biomass tended to have faster mineralization at 5–15 cm in invaded plots. These findings suggest that more attention to reference conditions is needed to understand the impact of invasive species on soil nitrogen cycling and other ecosystem processes and that the greatest impacts will not necessarily be where the invader is most abundant.

## Introduction

Invasive plants can alter ecosystem processes, but our ability to predict where those impacts will be most severe in an invaded landscape is limited. For example, invasive species can alter soil biogeochemical processes by changing litter inputs (Liao et al. 2008), the timing and location of nitrogen (N) released from decomposing litter (Laungani and Knops 2009), soil N transformations (Asner and Beatty 1996, Hawkes et al. 2005), and soil carbon losses (Litton et al. 2008, Strickland et al. 2010, 2011). The magnitude and direction of invader impacts on these processes can vary widely between sites (Warren et al. 2012). To better understand spatial variation in invader impacts, recent studies have investigated how environmental factors mediate the effects of invasions (Hulme et al. 2013, Castro-Díez et al. 2014, Lee et al. 2016). This effort should be extended to consider how environmental factors directly, indirectly, and interactively mediate invasion impacts on soil processes.

An invader can have variable impacts across a heterogeneous landscape through multiple pathways that vary in strength and direction. On the one hand, synergies among environmental conditions and invasive species’ abundance can occur if conditions that promote the species’ abundance also promote the magnitude of its ecosystem impact. On the other hand, the direction of these pathways can oppose each other such that it is difficult to predict where in the landscape, invasion impacts will be most severe. For example, the invasive grass *Microstegium vimineum* (stiltgrass, hereafter *M.v*.) is reported to have litter that promotes nitrification and benefits from nitrate nutrition (Lee et al. 2012) while being highly productive in wet habitats (Warren et al. 2010). We may expect (1) higher soil moisture content to directly inhibit nitrification by increasing the density of anoxic microsites and (2) moisture to indirectly promote nitrification by increasing nitrate availability and promoting invader biomass. In sum, it is difficult to know whether this invader’s impact on nitrification will be greatest where the invader is most productive in moist soils, or where the invader is less productive in drier soils. Knowledge of the relative importance of the pathways through which environmental factors, for example, soil moisture, can mediate the impact of invasion on soil N pools and fluxes, for example, nitrification rates, is necessary to predict where an invasion is likely to cause large changes. This knowledge is critical because plant invasions contribute to causing larger N pools and faster N fluxes (Lee et al. 2016), which are conditions that lead to plant communities with more invasive and fewer native taxa (Davis et al. 2000).

**Fig. 1.**
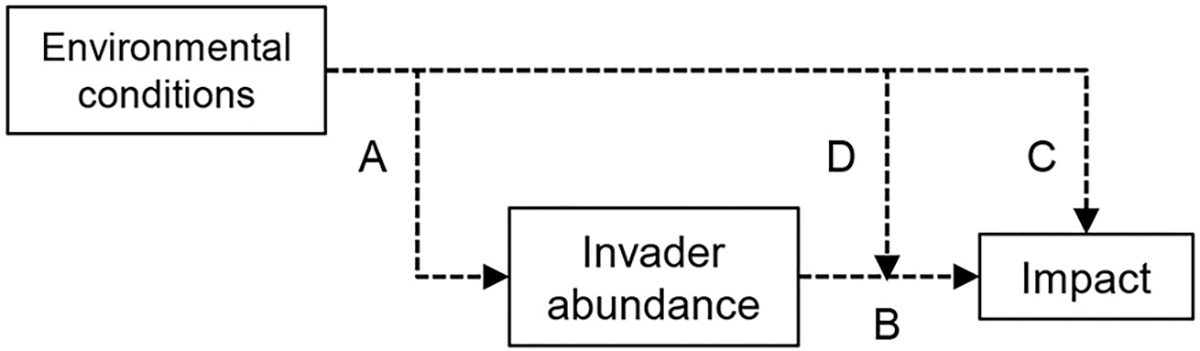
Environmental conditions can directly and indirectly mediate the magnitude and direction of invasion impacts on ecosystem processes. Environmental conditions can shape invader abundance (A), which can in turn determine the magnitude of an invasion impact (B). Environmental conditions can also directly modify the magnitude of an impact (C) and mediate the relationship between an invader’s abundance and its impact (D).

We propose a generalizable framework that outlines the roles of environmental factors in invasion impacts via a network of directed pathways (Fig. 1). First, an important component of a species’ effect on an ecosystem process is simply its dominance in the community (Parker et al. 1999, Grime 2001). In other words, we expect sites with more *M.v*. biomass to have relatively greater nitrate concentrations and net nitrification rates in invaded plots (Fig. 1, arrow A). By controlling invader abundance, environmental factors can indirectly mediate invasion impacts (Fig. 1, arrows A and B). For example, we expect that site-level environmental factors such as wet soils and high light availability will promote *M.v*. abundance (Adams and Engelhardt 2009, McGrath and Binkley 2009) and in turn promote invader impacts (Fig. 1, arrows A and B). Environmental conditions can also control the magnitude of an invader’s ecosystem impact in ways that are independent (Fig. 1, arrow C) and dependent upon invader biomass (Fig. 1, arrow D) by altering nutrient availability and microbial activity. We may expect a plant species with litter that tends to promote N mineralization (e.g., high N content and low C:N ratio) to have a smaller impact on N mineralization in high soil organic matter conditions since microbes are more limited by N and thus are more likely to immobilize it (Sterner and Elser 2002). Alternatively, this type of plant litter may have a larger impact on N mineralization in high soil organic matter conditions if the more labile plant inputs prime microbial activity to break down older stores of soil organic matter (Kuzyakov 2010). Understanding these processes as a generalizable network will help identify mechanisms through which environmental conditions mediate invasion impacts and determine which mechanisms are most influential.

Here, we use the invasive grass *M.v*. to investigate pathways through which environmental conditions mediate invader impacts on soil N cycling. *M.v*. is a widespread invader in the eastern United States (Stricker et al. 2016), and predicting its influences on soil biogeochemistry is necessary for managers working to control *M.v*. and other invasive plant species. Our hypothesis is that *M.v*. elevates inorganic N pools and fluxes, specifically nitrate concentrations and net nitrification rates (Kourtev et al. 1998, 2003, Ehrenfeld et al. 2001, Lee et al. 2012). While *M.v*. has been observed to promote soil nitrate concentrations and nitrification rates in a controlled common garden study (Lee et al. 2012), these effects may be highly variable across heterogeneous sites. Studies in forest uplands have reported that invaded plots do not differ from reference plots in inorganic N concentrations or potential N transformation rates (Adams and Engelhardt 2009, McGrath and Binkley 2009), whereas invasions in wet sites have found *M.v*. associated with elevated inorganic N concentrations (Ehrenfeld et al. 2001, DeMeester and Richter 2010).

We conducted our study across a series of invaded sites that varied in *M.v*. abundance and environmental conditions in forests located in Durham, North Carolina, USA, to ask: (1) What is the overall impact of *M.v*. presence on soil N pools and fluxes across the landscape? (2) How are site-level impacts mediated by *M.v*. biomass and reference environmental conditions, for example, soil moisture and organic matter, light availability, and understory vegetation? (3) What is the relative importance of direct, indirect, and interactive pathways that reference environmental conditions control the impact of *M.v*. presence on soil N pools and fluxes? We generated overall impact estimates to compare mean effect sizes and variability with other studies (Kourtev et al. 1998, 2003, Ehrenfeld et al. 2001, Adams and Engelhardt 2009, DeMeester and Richter 2010). Regarding the direction and relative strength of pathways that are independent of and dependent on *M.v*. biomass, little is known. Thus, our findings will help identify where an invasion is likely to cause large changes in soil N pools and fluxes.

## Methods

### Study design

In 2011, we established 16 sites on the edges of *M.v*.-invaded areas within Duke Forest, Durham, North Carolina, USA. Duke Forest is 2800 ha of up to 70-yr-old stands dominated by *Liquidambar styracifula, Liriodendron tulipifera, Quercus* spp., and *Pinus taeda. M.v.* has a patchy distribution across both upland and lowland forests in the Duke Forest. Like many plant invasions, the history of establishment in the area is uncertain but is often the result of natural and anthropogenic disturbances that create forest openings and seed movement along roads and waterways (Cole and Weltzin 2004, Anderson et al. 2012). Sites were only established at locations where there were no obvious environmental gradients (e.g., light availability or vegetation cover) to explain the presence or absence of *M.v*. Most sites were located on shallow Alfisol soils (Hapludalfs and Kanhapludalfs) having lithic contact within 50 cm of the mineral soil surface. Soils at five of the 16 sites were oxyaquic, whereas others were well drained and three of the 16 sites were kaolinitic. Soil textures ranged from coarse to fine sandy loam (USDA Soil classification database 2018).

Each study site consisted of a *M.v*.-invaded and a reference plot that were positioned to sample a six-meter transect across the invasion boundary. During peak growing season, August in 2012 and 2013, the three 0.25 × 0.25 m quadrats were aligned 1 m apart and perpendicular to the invasion boundary as in Warren et al. (2010; Appendix S1: Fig. S1). From each quadrat, we harvested vegetation, collected plant litter, and sampled soil in a similar manner as in Lee et al. (2012); see below for details.

### Site trees and understory light availability

In 2012, trees at each site were surveyed in within a 10 m radius centered on the 2012 transect invasion boundary. Only trees with greater than three centimeters diameter at breast height were included. With this information, we calculated the number of trees per site and the tree basal area per site (m^2^). In 2012, we determined the understory light availability of each plot. Light measurements were collected at the same time as the soil and vegetation harvest. Photosynthetically active radiation at one meter above each quadrat was measured and then averaged by plot. Reference light measurements outside of the forested sites and in full light were collected to calculate the percent of light reaching the understory at each site plot (AccuPAR Linear PAR/LAI ceptometer; Decagon Devices, Pullman, Washington, USA).

### Vegetation collection

We clipped all live herbaceous stems at the soil surface, sorted it into *M.v*. and non-*M.v*. plant material, and then collected and cleared plant litter in the quadrat to expose the soil organic layer. To standardize litter removal, we used clippers to cut the litter layer mat along the quadrat border and stopped removing litter from the soil surface when more soil was being removed than litter. Harvested plant material was dried and weighed in the laboratory.

### Soil collection

Immediately after collecting plant material, one soil core was collected (0–15 cm depth, 5 cm diameter) from the center of each quadrat (three quadrats per plot), split by depth (0–5, 5–15 cm), and then pooled by plot. Soils were separated by depth to account for higher soil organic matter in the 0–5 cm layer. No obvious differences in earthworm presence were detected among the samples. The *M.v*. rooting zone is generally shallow but can reach depths of at least 15 cm (DeMeester 2009, Warren et al. 2010). Soils were sieved through a 2-mm mesh and within 48 h of field sampling soil inorganic N was extracted with 2M KCl (10:1) and measured with an autoanalyzer (Lachat, Hach, Loveland, Colorado, USA). Net N mineralization rates were estimated as in Lee et al. (2012) by incubating soils aerobically in the laboratory at 22°C for 12–14 d at field moisture levels. We did not adjust soil moisture prior to the incubations so that we could capture the site differences in soil moisture in this measure. Incubating soils were covered in punctured Parafilm to minimize soil moisture loss while allowing for aeration. Soil moisture was measured gravimetrically. Soil inorganic N concentrations were transformed to represent μg of N per gram of dry soil (hereafter μgN/G). Inorganic N measurements below analytical detection (0.002 ppm for ammonium and 0.006 ppm for nitrate) were represented with the value 0.0001 μgN/G. Net ammonification, nitrification, and mineralization were calculated as the difference in ammonium, nitrate, and total inorganic N (μgN/G), respectively, at the end and start of the incubation, divided by the number of incubation days. Ammonification, nitrification, and mineralization rates have units of μgN/G per day (hereafter μgN/G × d). The percentage of soil organic matter was determined by loss on ignition using a muffle furnace at 430°C. Soils were not evaluated for carbonates which can bias soil organic matter estimates, so our measure (hereafter referred to simply as “soil organic matter”) is a reflection of organic matter and carbonates.

### Statistical analysis—mean invasion impacts

To determine whether soil N pools and fluxes differed between paired invaded and reference plots across all sites, we ran general linear mixed-effects models (GLMMs) using invasion status as a fixed effect and site and year as random effects. A total of 10 soil N pools and flux measures were evaluated: ammonium (μgN/G), nitrate (μgN/G), ammonification (μgN/G × d), nitrification (μgN/G × d), and mineralization (μgN/G × d) at 0–5 and 5–15 cm soil depths.

### Statistical analysis—site-level predictors of invasion impacts

Our goal was to determine how reference environmental conditions and *M.v*. abundance mediate the impact of *M.v*. invasion on soil nitrate concentrations and nitrification and mineralization rates. We also chose to investigate any soil N pool or flux that responded to invasion status in the previous analysis. To quantify the impact of *M.v*. invasion, we calculated the difference between each paired invaded and reference plot value (i.e., Invaded – Reference) at 0–5 and 5–15 cm. Hereafter, these difference quantities are presented as Δ (e.g., Δ nitrification at 0–5 cm). Values greater than zero indicate an increase in the invaded plot relative to the reference plot.

To determine how reference environmental conditions and *M.v*. abundance mediate Δ soil N pools and fluxes, we evaluated GLMMs with reference variables, *M.v.* biomass, and their interactions. Since we measured many reference variables and relatively few sites (*n* = 16, sampled in 2012 and 2013), we used two model selection procedures to identify the best subset of reference variables to explain each Δ soil N pool and flux.

First, we built a full model for each Δ response variable with year as a random effect and fixed effects that included all possible reference variables and *M.v*. biomass; no interaction terms were included. Reference variables included reference plot soil measures (i.e., N pools, N fluxes, organic matter, and pH), reference plot vegetation measures (i.e., understory and litter biomass), and site tree measures (i.e., number of trees and tree basal area). For each Δ response variable, select reference variables were excluded as predictors to avoid circularity (see Appendix S1: Table S1), and variables from a single soil depth were used to explain changes in that same soil depth (i.e., multiple soil depths were not included in the same model). Each full model was compared to an intercept-only model using analysis of variance to determine whether any of the reference variables and/or *M.v*. biomass terms provided useful information about any of the Δ soil N pools or fluxes (Table 1). For the Δ response variables that were better explained by the full model than the intercept-only model, we used step-wise model selection to identify the best combination of reference variables. We forced the *M.v*. biomass term to be retained in all models. Step-wise model selection on GLMMs was performed using the R package lmerTest and the function step.

Next, we added *M.v*. biomass interaction terms to individual reference variables that were retained in each model and used step-wise model selection to remove the unnecessary interaction terms. In sum, the model selection steps resulted in a final model for each Δ response variable that included (1) *M.v*. biomass, (2) select reference variables, and (3) select interaction terms between *M.v*. biomass and reference variables as fixed effects and year as a random effect.

### Statistical analysis—site-level predictors of *M.v.* abundance

To determine how reference environmental conditions might indirectly mediate Δ soil N pools or fluxes through *M.v*. abundance, we needed to identify a model that uses reference variables to explain *M.v.* biomass. To do this, we built a full model with year as a random effect and fixed effects that included all possible reference variables, averaged by soil depth. Possible reference variables included reference plot soil measures (except N fluxes), reference plot vegetation measures, light availability, and site tree measures. Reference N fluxes were excluded as predictors to avoid circularity in the final structural equation model, described below.

### Statistical analysis—structural equation modeling

We used piecewise structural equation modeling (SEM) to link the final GLMMs obtained through model selection in a causal network (Shipley 2002). Model fit was evaluated based on the Fisher’s C statistic and a chi-square distribution significance test. Standardized path coefficients and marginal *R*^2^ values (Nakagawa and Schielzeth 2012) were reported from model output (Lefcheck 2016). The strength of a compound path (i.e., indirect effect) is the product of the coefficients along the path. The total effect of one variable on another equals the sum of its direct and indirect effects.

All analyses were conducted using R Statistical Software (R Core Team 2016). For all analyses with mixed-effects models, we used the lme4 and lmerTest packages to fit the models, calculate degrees of freedom with Satterthwaite approximations, and ran t-tests to evaluate whether fixed effect coefficients significantly differed from zero (Kuznetsova et al. 2014, Bates et al. 2015). Piecewise SEM was conducted using the piecewiseSEM package (Lefcheck 2016).

## Results

### Mean invasion impacts

Invaded plots had faster ammonification rates at 0–5 cm (*P* = 0.01) and marginally faster mineralization rates at 5–15 cm (*P* = 0.07; Appendix S1: Fig. S2 and Table S2). These trends were largely driven by differences between invaded and reference plots in 2013. Nitrate concentrations were lower in 2013 than 2012 (Appendix S1: Fig. S2). Ammonium and nitrate concentrations were consistently higher at 0–5 than 5–15 cm (Appendix S1: Fig. S2). For the following analyses of drivers of site-level invasion impacts, we did not investigate ammonium concentrations because we did not detect a mean effect of invasion on ammonium concentrations across sites, and there is no previous evidence that ammonium concentrations respond to the presence of *M.v*.

### Key reference conditions that mediate invasion impacts and M.v.abundance

We tested whether site-level Δ nitrate, Δ ammonification, Δ nitrification, and Δ mineralization at 0–5 and 5–15 cm could be explained by reference variables (see Appendix S1: Table S1 for list of variables used in each model to avoid circularity) and *M.v*. biomass. These variables could not explain Δ nitrate, but were useful for understanding Δ ammonification (5–15 cm), Δ nitrification (0–5 cm), and Δ mineralization (0–5, 5–15 cm; Table 1, all *P* < 0.01).

We used model selection to reduce the number of reference variables and found that models with reference soil nitrate and moisture best explained Δ nitrification and Δ mineralization at 0–5 cm (Appendix S1: Table S3, Model selection 1: Reduce reference variables). Significant effects of nitrate on Δ nitrification (0–5 cm) and nitrate and soil moisture on Δ mineralization (0–5 cm) should be considered with caution since the sample with the highest nitrate and moisture is very influential (see Appendix S1: Fig. S5). The addition of interaction terms between each reference variable (i.e., soil nitrate and moisture) and *M.v*. biomass did not improve model fit for Δ nitrification or Δ mineralization at 0–5 cm (Appendix S1: Table S3, Model selection 2: Add interactions).

For Δ ammonification and Δ mineralization at 5–15 cm, reference variables included in the best models were understory biomass and soil nitrate (Appendix S1: Table S3, Model selection 1: Reduce reference variables). In addition, tree basal area was included in the best model for Δ ammonification at 5–15 cm (Appendix S1: Table S3). The addition of an interaction term between reference soil nitrate and *M.v*. biomass improved each model for Δ ammonification and Δ mineralization at 5–15 cm (Appendix S1: Table S3, Model selection 2: Add interactions).

Light availability and tree basal area were included in the best model to explain variation *M.v*. biomass (Appendix S1: Table S4).

### Direct, indirect, and interactive effects of reference conditions

**Fig. 2.**
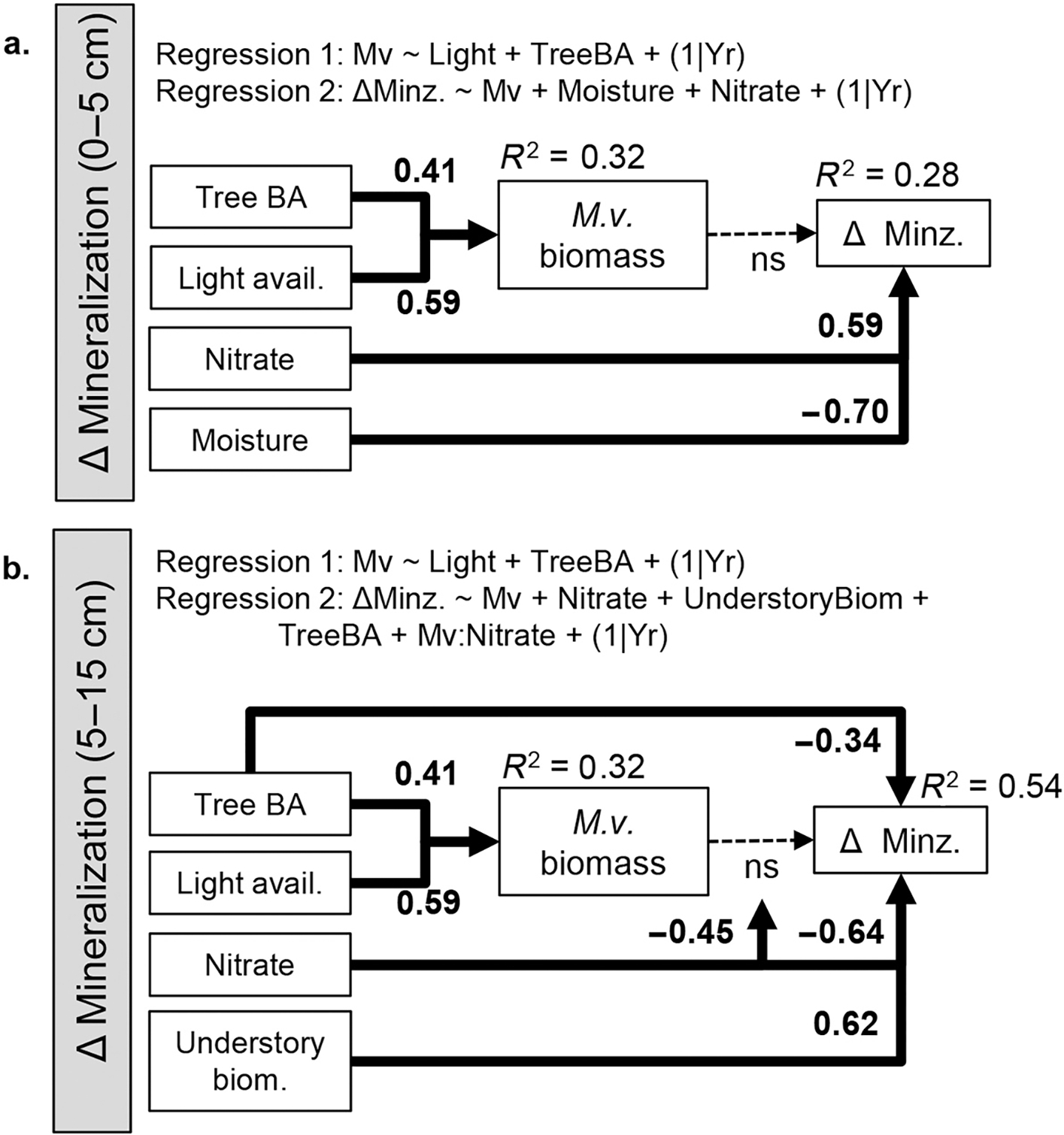
Environmental conditions including tree basal area, light availability, soil nitrate, soil moisture, and understory biomass mediate the impact of *M.v*. invasion on mineralization at 0–5 and 5–15 cm through indirect, direct, and interactive pathways. Piecewise structural equation models were constructed for Δ mineralization (D= Inv. – Ref.) at each soil depth using regressions 1 and 2. The data adequately fit each model (C = 7.21, 10.22; df = 8, 6; *P* > 0.05). Values on top of arrows indicate the standardized path coefficient and solid bold arrows are significant (alpha = 0.05); marginal *R*^2^ values are provided. An arrow that points to another arrow illustrates an interaction term (e.g., Fig 1, arrow D). Tree BA is shorthand for “Tree basal area.” Path diagrams built to understand Δ nitrification (0–5 cm) and Δ ammonification (5–15 cm; Appendix S1: Fig. S2) are analogous to the mineralization results presented here. Significant paths between nitrate, moisture, and Δ mineralization (0–5 cm; a) should be considered with caution since the highest nitrate and moisture sample is very influential (see Appendix S1: Fig. S5).

Piecewise SEM models were constructed using the final regression models (Fig. 2; Appendix S1: Tables S3, S4, Fig. S3). SEM models for Δ nitrification at 0–5 cm (Appendix S1: Fig. S3a, C = 7.23; df = 8, *P* > 0.05), Δ mineralization at 0–5 cm (Fig. 2a, C = 7.21, df = 8, *P* > 0.05), and Δ ammonification at 5–15 cm (Appendix S1: Fig. S3b, C = 10.95, df = 6, P > 0.05) were adequately supported by the data. The initial SEM model for Δ mineralization at 5–15 cm did not fit the data (Appendix S1: Fig. S4; C = 18.34; df = 8; *P* = 0.02). To improve the model fit, tree basal area was added as a direct predictor of Δ mineralization at 5–15 cm since this term was marginally significant predictor (*P* = 0.09, Appendix S1: Table S3). The resulting SEM model for Δ mineralization at 5–15 cm was adequately supported by the data (Fig. 2b, C = 10.22, df = 6, *P* > 0.05). Differences in the rate of inorganic N transformations in invaded vs. reference plots were mediated by environmental conditions through different pathways at 0–5 and 5–15 cm soil depths.

At the 0–5 cm soil depth, Δ mineralization (Fig. 2a) and Δ nitrification (Appendix S1: Fig. S3a) were directly mediated by reference soil nitrate and moisture; however, again, significant effects of nitrate on Δ nitrification (0–5 cm) and nitrate and soil moisture on Δ mineralization (0–5 cm) should be considered with caution since the sample with the highest nitrate and moisture is very influential (see Appendix S1: Fig. S5). Soil nitrate was positively correlated with Δ mineralization (std. coef. = 0.59; Fig. 2a) and Δ nitrification (std. coef. = 0.58, Appendix S1: Fig. S3a) at 0–5 cm such that sites with nitrate >2 μgN/G were more likely to experience increases in invaded plots (Appendix S1: Fig. S5). Soil moisture was negatively correlated with Δ mineralization (std. coef. = 0.70, Fig. 2a) and Δ nitrification (std. coef. = 0.65, Appendix S1: Fig. S3a) at 0–5 cm such that sites with moisture <20% were more likely to experience increases in nitrification and mineralization in invaded plots (Appendix S1: Fig. S5). The absolute influence of reference moisture was slightly stronger than that of nitrate on Δ mineralization and Δ nitrification at 0–5 cm. There was no evidence that *M.v*. biomass increased the difference in mineralization or nitrification in invaded relative to reference plots (Fig. 2a; Appendix S1: Fig. S3a).

At the 5–15 cm soil depth, Δ mineralization (Fig. 2b) and Δ ammonification (Appendix S1: Fig. S3b) were mediated by environmental conditions through multiple pathways types.

**Fig. 3.**
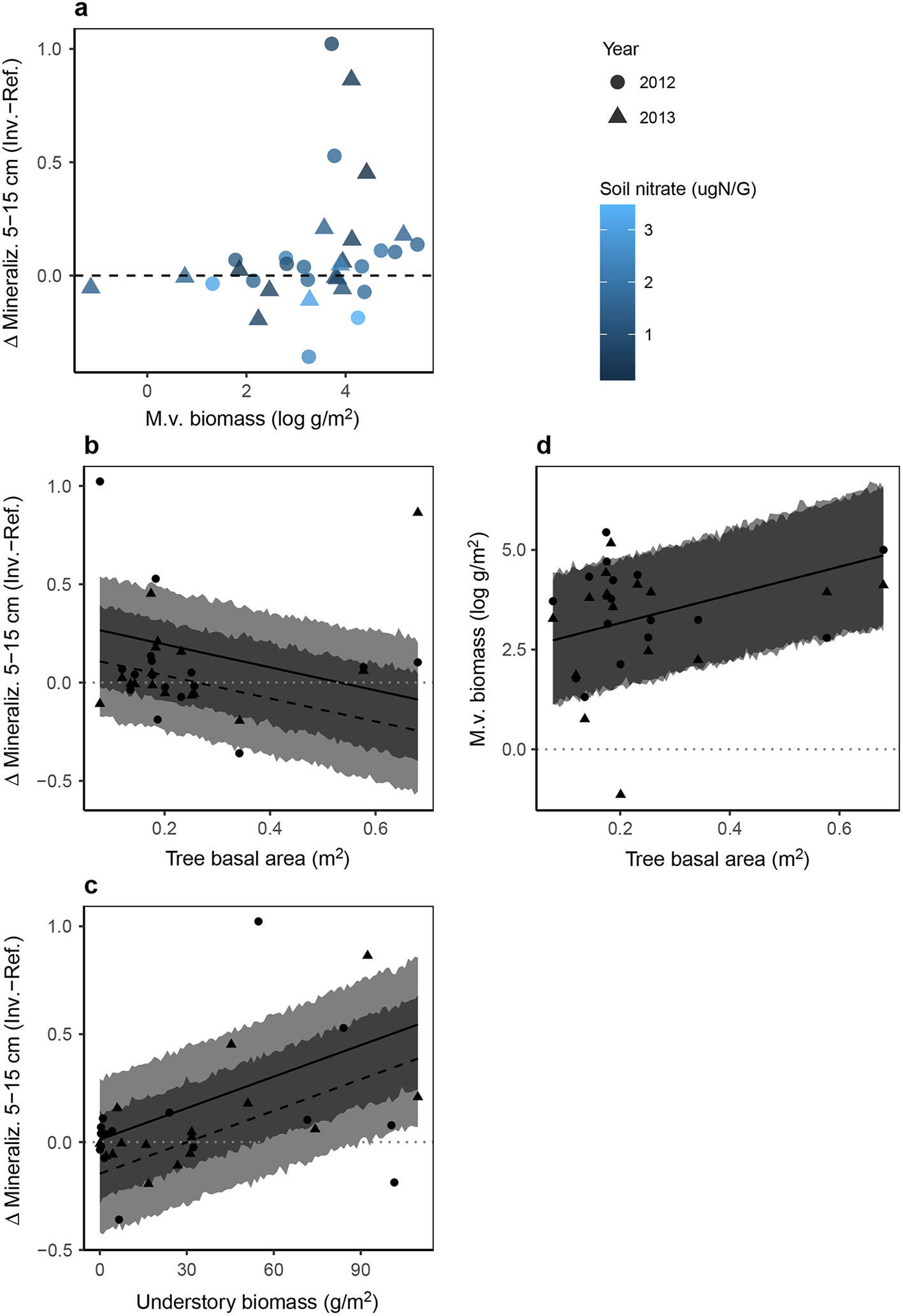
Mineralization (5–15 cm) differences between paired reference and invaded plots are explained by (a) an interaction between *M.v*. biomass and reference soil nitrate, (b) tree basal area, and (c) understory biomass (Appendix S1: Table S3). Although tree basal area (d) promotes *M.v*. biomass (Appendix S1: Table S4), it (b) diminishes the impact of *M.v*. on mineralization at 5–15 cm. Patterns associated with Δ ammonification at 5–15 cm are analogous (Appendix S1: Fig. S5). Each point represents a site (*n* = 16) and year. For panel a, lighter point color represents larger soil nitrate concentrations. For panels b–d, model fits and 95% prediction intervals are shown conditional on year (2012 = solid line, 2013 = dotted line; prediction intervals overlap in this figure).

First, *M.v*. biomass and reference soil nitrate had an interactive effect on Δ mineralization (std. coef. = −0.45, Fig. 2b) and Δ ammonification at 5–15 cm (std. coef. = −0.46; Appendix S1: Fig. S3b). These inorganic *N* fluxes only were greater in invaded plots in the presence of high *M.v*. biomass (>20 g/m^2^) and low reference soil nitrate concentrations (<2 μgN/G; Fig. 3a; Appendix S1: Fig. S6a).

Second, tree basal area and understory biomass directly influenced Δ mineralization and Δ ammonification at 5–15 cm. Tree basal area was negatively associated with Δ mineralization (std. coef. = 0.34, Figs. 2b, 3b) and Δ ammonification (std. coef. = 0.47, Appendix S1: Figs. S2b, S6b), whereas understory biomass was positively associated with Δ mineralization (std. coef. = 0.62, Figs. 2b, 3c) and Δ ammonification (std. coef. = 0.60, Appendix S1: Figs. S3b, S6c).

Third, light availability and tree basal area had a weak indirect influence on Δ mineralization and Δ ammonification by promoting *M.v*. biomass (Fig. 3d and Appendix S1: Fig. S7) that in turn could promote Δ mineralization and Δ ammonification at low-nitrate sites (Fig. 3a and Appendix S1: Fig. S6a). Trees block understory light availability so there were no sites with high light availability and high tree basal area (Appendix S1: Fig. S8a). Yet, sites with low light availability varied widely in tree basal area; conversely, sites with low tree basal area varied widely in light availability. Understory biomass increases with tree basal area (Appendix S1: Fig. S8b, *P* = 0.01) but was not related to light availability (Appendix S1: Fig. S8c). Understory biomass was not a strong predictor of invader biomass (Appendix S1: Fig. S9, Table S4).

Tree basal area had different direct and indirect roles on Δ mineralization and Δ ammonification. Whereas tree basal area directly diminished Δ mineralization (std. coef. = −0.34, Fig. 2b) and Δ ammonification (std. coef. = 0.47, Appendix S1: Fig. S3b) at 5–15 cm, it indirectly promoted Δ mineralization (path strength = 0.41 × 0.17 (ns) = 0.07; Fig. 2b) and Δ ammonification (path strength = 0.41 × 0.27 = 0.11; Appendix S1: Fig. S3b) by promoting *M.v*. biomass. In sum, tree basal area had a net negative influence on Δ mineralization (−0.34 [direct] + 0.07 [indirect] = −0.27, Fig. 2b) and Δ ammonification (−0.47 [direct] + 0.07 [indirect] = −0.40; Appendix S1: Fig. S2b).

Overall, the strongest pathways acting on Δ mineralization and Δ ammonification at 5–15 cm were (1) the direct influence of nitrate (−0.64, −0.56) and (2) the direct influence of understory biomass (0.62, 0.60), followed by (3) the interactive influence of nitrate and *M.v*. biomass (−0.45, −0.46) and (4) the direct influence of tree basal area (−0.34, −0.47), and last (5) the indirect influence of tree basal area (0.07, 0.11; std. coefs from Fig. 2b and Appendix S1: Fig. S3b).

## Discussion

Through an in-depth study of how reference environmental conditions mediate the impacts of an invasive grass on soil N cycling, we see that reference conditions can (1) be highly influential, (2) mediate impacts through multiple pathways, and (3) have very different effects on N cycling at different soil depths. Unlike previous studies, we did not find an overall pattern of elevated nitrate concentrations or higher nitrification rates in *M.v*.-invaded areas (Appendix S1: Fig. S2). We found that reference plot conditions mediated differences in mineralization between paired invaded and reference plots at each site through indirect (via *M.v.* biomass), direct, and interactive pathways; however, the strongest pathways were independent of *M.v*. biomass (Fig. 2). Reference conditions, including nitrate concentrations, soil moisture, understory biomass, and tree basal area, were particularly influential (Fig. 3, Appendix S1: Figs. S5, S6). Detailed studies that incorporate experimental manipulations will be necessary to understand the mechanisms by which these factors influence the apparent impact of *M.v*. on N mineralization. In sum, the greatest impacts of the invasive grass on soil N pools and fluxes may not necessarily be where the invader is most abundant.

We hypothesized that sites with more *M.v*. biomass would have relatively higher nitrate concentrations and net nitrification rates in invaded plots (Fig. 1, arrow A), but we did not observe an effect of *M.v*. presence on nitrate or nitrification. In addition, we did not detect a strong role of *M.v.* biomass on Δ N pools or fluxes (Δ = Invaded – Reference) across sites. There are a couple of potential reasons why our study did not detect these patterns. First, paired plots were very close to one another (2 m apart) in this study. The purpose of this design was to maximize the likelihood that reference plot conditions were as close a representation as possible to conditions in the invaded plot prior to the presence of *M.v*. However, this design also meant that invaded plots sampled the edges of *M.v*. stands.

*M.v*. at stand edges can be relatively low density and indeed, less than half of the invaded plots in our study had more than 50% *M.v*. cover and we observed densities of approximately 50 g/m^2^. Previous studies that have observed elevated nitrate and nitrification beneath *M.v*. observed average densities ranging from 42 and 138 g/m^2^ (Ehrenfeld et al. 2001) to 553 and 725 g/m^2^ (DeMeester and Richter 2010). Thus, *M.v*. densities in this study are on the low end of the range found to impact nitrification rates, which may explain why we did not observe the expected soil N impacts.

*M.v*. at stand edges also may not have been established for very long. It can take more than one growing season to observe the influence of a plant species on soil N pools and fluxes, especially if litter inputs are important. While *M.v*. impacts on soil nitrate and nitrification may be the consequence of above- and/or belowground litter inputs, these effects have been observed by experimentally growing *M.v*. in greenhouse conditions in as little as three months (Lee et al. 2012). In sum, a too short of a time-since-invasion in this study’s invaded plots is a potential, but unlikely rationale for why no typical invasion impacts were observed.

Another potential reason why we did not observe elevated nitrate or nitrification in invaded plots or in plots with greater *M.v*. densities is that soil pH was relatively low across all our study sites. Many of our sites had acidic soils and were in forested areas in the presence of pine trees or former pine plantations, whereas previous studies were in different plant communities or types of land use (Kourtev et al. 1999, Ehrenfeld et al. 2001, Lee et al. 2012). The activity of autotrophic nitrifying bacteria is inhibited in low pH soil conditions (De Boer and Kowalchuk 2001), and this process may have contributed to the absence of an effect of *M.v*. presence on nitrification (Shannon-Firestone et al. 2015). In addition, low ammonium availability at our sites (~2–3 μgN/G, Appendix S1: Fig. S2) may not support nitrifier communities that drive nitrification. Reference ammonium pools in Ehrenfeld et al. (2001) were much higher (~10 μgN/G). However, ammonium concentrations were approximately equivalent in Lee et al. (2012; field study ~2 lgN/G and common garden ~2–3 μgN/G), and nevertheless, strong *M.v*. impacts on nitrate and nitrification were detected. Although we did not detect a strong role of *M.v*. biomass on Δ N pools or fluxes, we did find that reference plot conditions including nitrate, moisture, understory biomass, and tree basal area were important in mediating Δ mineralization rates.

Reference soil nitrate was an important regulator of N mineralization differences between paired invaded and reference plots but direction of the relationship differs in the 0–5 and 5–15 cm layer (Fig 2). That being said, significant effects of nitrate on Δ nitrification (0–5 cm) and Δ mineralization (0–5 cm) should be considered with caution since the sample with the highest nitrate and moisture was very influential (see Appendix S1: Fig. S5). Assuming the pattern at 0–5 cm is representative rather than spurious, higher reference nitrate concentrations at 0–5 cm are associated with greater nitrification and mineralization in invaded plots, whereas lower reference nitrate concentrations at 5–15 cm are associated with higher ammonification and mineralization. Low nitrate in reference soils can suggest that N release from organic matter is slow relative to plant and microbial N uptake and/or that soil texture properties permit nitrate to be quickly leached from the soil profile. These conditions may promote the immobilization of N in invaded plots relative to reference plots (e.g., at 0–5 cm) if newly available inorganic N in invaded plots is quickly immobilized by historically N-limited microbes. Alternatively, these conditions may promote mineralization in invaded plots (e.g., at 5–15 cm) labile *M.v*. inputs prime N release. The finding that ammonification and mineralization only were greater at sites with high *M.v*. density supports this idea (Fig. 3a). The mechanisms underlying these relationships will need to be studied with experimental manipulation and in situ mineralization measures.

Sites with reference soil moisture less than approximately 20% were more likely to experience greater nitrification and mineralization in invaded plots (Appendix S1: Fig. S5). This pattern may be a consequence of numerous anoxic microsites within moist soils and periodic inundation at oxyaquic sites. Another explanation is that soil moisture may act as a proxy for unmeasured differences in soil texture that are important in mediating microbial N transformations.

Sites with more understory vegetation in reference plots tended to have faster mineralization and ammonification at 5–15 cm in invaded plots (Fig. 3c, Appendix S1: Fig. S6c). One reason for this pattern could be that sites with depauperate understories are disturbed by deer grazing and thus have relatively high mineralization rates prior to invasion. Non-*M.v*. understory vegetation was relatively sparse across sites (mean SE: reference = 32.26 6.28 g/m^2^, invaded = 33.47 8.93 g/m^2^), and invaded plots had on average about double the total understory vegetation relative to reference plots (mean SE: reference = 32.26 6.28, invaded = 84.89 14.11). However, examination of the reference plot mineralization data does not support this hypothesis. Instead, sites with dense reference understories may include species with root and/or litter inputs that interact with *M.v*. inputs to prime mineralization. Sites with dense understory vegetation included grasses and sedges, whereas more sparsely vegetated sites typically consisted of native and non-native vines (i.e., *Vitus* spp., *Lonicera japonica*) and tree seedlings. Additional investigation into the understory community compositional shifts at the study sites could provide insight into the pattern we observed that reference sites with dense understory vegetation tended to experience greater mineralization in the presence of *M.v*.

Tree basal area had a relatively unexpected indirect and direct impact on Δ mineralization and Δ ammonification at 5–15 cm (Fig. 2b, Appendix S1: Fig. S3b). We hypothesized that tree basal area would be negatively correlated with understory light availability, which would act to limit *M.v*. biomass. In addition, we anticipated that greater tree basal area might mediate inorganic N demand and directly affect *M.v*. impacts on soil N pools and fluxes. We found that light availability was positively associated with *M.v*. biomass, but that light availability was not entirely dependent on tree basal area. Both *M.v*. biomass and trees basal area were associated with greater *M.v*. biomass. The mechanism underlying the positive association between tree basal area and *M.v*. biomass is unclear, but may relate to particular species interactions between *M.v*. and tree species at sites with high tree basal area, which tend to be fast-growing early-successional species (site 9 = *Acer-Liquidambar-Liriodendron* and site 2 = *Liquidambar-Pinus*). In addition, tree basal area may be a proxy for important but unmeasured variables such as land use history and soil texture; however, soil classifications at sites with high tree basal area did not differ.

In contrast to the weak indirect positive influence of tree basal area on Δ mineralization and Δ ammonification at 5–15 cm via *M.v*. biomass (and only at sites with low-nitrate reference soils), this variable had a direct negative association with Δ mineralization and Δ ammonification at 5–15 cm. In other words, sites with low tree basal area were more likely to experience greater mineralization, especially ammonification, in invaded plots at 5–15 cm (Fig. 3b, Appendix S1: Fig. S6b). A potential explanation for this pattern is that sites with low tree basal area had less active root biomass at 5–15 cm so that *M.v*. presence stimulated rhizosphere processes, including microbial-mediate mineralization, to greater extents at these sites.

Our study provides evidence that consideration of environmental context is critical for understanding where, and under what conditions, the impacts of an invasive plant species will occur. We show that environmental factors can influence the direction of relationships between invader abundance and impact. Moreover, environmental conditions can mediate invader impacts directly and indirectly. This view of invader impacts allows us to detect where environmental factors that promote invader abundance can also limit its impacts at a given site. Although more research is needed to experimentally test the causal links supported by the data we have presented, we demonstrate that the direct influence of site conditions can be more important than invader abundance in explaining invasive plant impacts on soil nitrogen cycling.

## Supplementary Material

supplement

## Figures and Tables

**Table 1.** Analysis of variance summary compares full and intercept-only models to explain site-level invasior impacts on nitrate, ammonification, nitrification, and mineralization (Δ = Inv. - Ref.).

Response variable	Model type	0–5 cm				5-15 cm			
	
		df	AIC	Chisq	*P* value	df	AIC	Chisq	*P* value

Δ Nitrate	~1	3	98.27			3	80.68		
	~ref vars + Mv biomass	14	111.21	9.06	0.6165	14	93.80	8.88	0.6333
Δ Ammonif.	~1	3	−0.33			3	−6.92		
	~ref vars + Mv biomass	14	2.81	18.85	0.0638	14	−15.99	31.07	**0.0011**
Δ Nitrif.	~1	3	36.12			3	−17.48		
	~ref vars + Mv biomass	14	30.40	27.72	**0.0036**	14	−7.95	12.47	0.3294
Δ Mineraliz.	~1	3	37.90			3	13.85		
	~ref vars subset + Mv biomass	13	32.93	24.97	**0.0054**	13	4.81	29.03	**0.0012**

*Notes:* Full models include all possible reference variables and *M.v.* biomass. Reference variables that are used to calculate the response variable are not included as predictors; see Appendix S1: Table S2 for details. Year is included as a random effect in all models. Fixed effect coefficients that significantly differ from zero are bold (alpha = 0.05).

## Data Availability

Data and code are publically available at https://github.com/marissalee/E8-NichePlots.
