# Supplementary material for: Site conditions are more important than abundance for explaining plant invasion impacts on soil nitrogen cycling
Source: Ecosphere. Author manuscript; Available in PMC 2024 Feb 14. (PMC10866306; doi:10.1002/ecs2.2454)

**Appendix S1**

Journal: Ecosphere
Manuscript title: Site conditions are more important than abundance for explaining plant invasion impacts on soil nitrogen cycling
Authors: Marissa R. Lee, S. Luke Flory, Richard P. Phillips, and Justin P. Wright

**Table S1.** List of reference environmental variables included in the full model to explain Δ nitrate, Δ ammonification, Δ nitrification, and Δ mineralization (Δ = Inv. – Ref.). An "x" indicates that the reference variable (rows) was included in the full model to explain the response variable (columns). Reference variables were excluded if they were used to calculate the response (red) or are highly correlated with the response (pink). Light availability was also excluded because it cannot directly affect N pools and fluxes (gray). Reference variables from a single soil depth were used to explain changes in that same soil depth, i.e. multiple soil depths were not included in the same model.

| Reference environment variable | Response variable | | | |
|---|---|---|---|---|
| | Δ Nitrate | Δ Ammonif. | Δ Nitrif. | Δ Mineraliz. |
| Ammonium | x | x | x | x |
| Nitrate | | x | x | x |
| Ammonif. | x | | x | |
| Nitrif. | x | x | | |
| Mineraliz. | x | | | |
| Moisture | x | x | x | x |
| SOM | x | x | x | x |
| pH | x | x | x | x |
| Light avail. | | | | |
| Understory biomass | x | x | x | x |
| Litter biomass | x | x | x | x |
| Number of trees | x | x | x | x |
| Tree basal area | x | x | x | x |

**Table S2.** Summary of the effects of M.v. invasion on soil N pools and fluxes. Plot type (invaded or reference) is the fixed effect for which estimates, standard error, degrees of freedom and p-values are shown. Random effects included site and year. Fixed effect coefficients that significantly differ from zero are bold (alpha=.05).

| Response variable | Plot | Year | 0-5cm | | | | 5-15cm | | | |
|---|---|---|---|---|---|---|---|---|---|---|
| | | | n | Mean | SE | p value | n | Mean | SE | p value |
| Ammonium | Invaded | 2012 | 16 | 3.06 | 0.62 | 0.60 | 16 | 1.88 | 0.17 | 0.28 |
| | | 2013 | 16 | 3.28 | 0.53 | | 16 | 2.24 | 0.27 | |
| | Reference | 2012 | 16 | 2.89 | 0.35 | | 16 | 2.18 | 0.34 | |
| | | 2013 | 16 | 3.04 | 0.45 | | 16 | 2.38 | 0.35 | |
| Nitrate | Invaded | 2012 | 16 | 3.3 | 0.29 | 0.10 | 16 | 1.82 | 0.28 | 0.94 |
| | | 2013 | 16 | 2.07 | 0.41 | | 16 | 1.14 | 0.16 | |
| | Reference | 2012 | 16 | 2.95 | 0.22 | | 16 | 1.89 | 0.17 | |
| | | 2013 | 16 | 1.8 | 0.36 | | 16 | 1.09 | 0.2 | |
| Ammonif. | Invaded | 2012 | **16** | **-0.02** | **0.03** | **0.01** | 16 | 0.01 | 0.04 | 0.10 |
| | | 2013 | **16** | **0.14** | **0.06** | | 16 | 0.05 | 0.05 | |
| | Reference | 2012 | **16** | **0.02** | **0.02** | | 16 | 0 | 0.02 | |
| | | 2013 | **16** | **-0.1** | **0.03** | | 16 | -0.06 | 0.03 | |
| Nitrif. | Invaded | 2012 | 16 | 0.15 | 0.04 | 0.36 | 16 | 0.13 | 0.05 | 0.28 |
| | | 2013 | 16 | 0.29 | 0.09 | | 16 | 0.16 | 0.05 | |
| | Reference | 2012 | 16 | 0.16 | 0.05 | | 16 | 0.05 | 0.02 | |
| | | 2013 | 16 | 0.42 | 0.09 | | 16 | 0.17 | 0.04 | |
| Mineraliz. | Invaded | 2012 | 16 | 0.14 | 0.08 | 0.60 | 16 | 0.14 | 0.03 | 0.07 |
| | | 2013 | 16 | 0.21 | 0.06 | | 16 | 0.43 | 0.08 | |
| | Reference | 2012 | 16 | 0.05 | 0.03 | | 16 | 0.18 | 0.04 | |
| | | 2013 | 16 | 0.12 | 0.04 | | 16 | 0.31 | 0.09 | |

**Table S3.** Analysis of variance summary from model selection procedures to explain Δ nitrification (0-5cm), Δ mineralization (0-5cm), Δ ammonification (5-15cm), and Δ mineralization (5-15cm) (Δ = Inv. – Ref.) using reference variables and M.v. biomass. Year is included as a random effect in all models. The first model selection procedure is to reduce the number of reference variables. The second model selection procedure is to add an interaction term with M.v. biomass to each reference variable retained from the previous selection procedure. M.v. biomass is always retained as a fixed effect. Fixed effect coefficients that significantly differ from zero are bold (alpha=.05). The significant nitrate and moisture terms for Δ nitrification (0-5cm) and Δ mineralization (0-5cm) should be considered with caution since the highest nitrate and moisture sample is very influential (see Fig. S5).

| Resp. var. | Model selection 1: Reduce reference variables | | | | | | | Model selection 2: Add interactions | | | | | | |
|---|---|---|---|---|---|---|---|---|---|---|---|---|---|---|
| | Term | SS | Num DF | Den DF | F value | Elim. | p value | Term | SS | Num DF | Den DF | F value | Elim. | p value |
| Δ Nitrif. (0-5cm) | Ammonif | 0.00 | 1 | 20.00 | 0.01 | 1 | 0.9133 | M.v. biomass : Nitrate | 0.04 | 1 | 25.00 | 0.35 | 1 | 0.5590 |
| | pH | 0.00 | 1 | 21.00 | 0.03 | 2 | 0.8545 | M.v. biomass : Moisture | 0.12 | 1 | 26.80 | 1.14 | 2 | 0.2956 |
| | SOM | 0.13 | 1 | 22.00 | 1.42 | 3 | 0.2459 | Nitrate | 1.07 | 1 | 18.74 | 10.45 | kept | 0.0044 |
| | Ammonium | 0.14 | 1 | 23.00 | 1.52 | 4 | 0.2303 | Moisture | 1.54 | 1 | 24.78 | 15.06 | kept | 0.0007 |
| | UnderstoryBiom | 0.12 | 1 | 23.75 | 1.26 | 5 | 0.2728 | M.v. biomass | 0.13 | 1 | 27.06 | 1.28 | kept | 0.2675 |
| | TreeBA | 0.10 | 1 | 24.30 | 1.13 | 6 | 0.2976 | | | | | | | |
| | LitterBiom | 0.15 | 1 | 25.71 | 1.58 | 7 | 0.2198 | | | | | | | |
| | Number of trees | 0.30 | 1 | 26.01 | 3.21 | 8 | 0.0849 | | | | | | | |
| | Nitrate | 1.07 | 1 | 18.74 | 10.45 | kept | 0.0044 | | | | | | | |
| | Moisture | 1.54 | 1 | 24.78 | 15.06 | kept | 0.0007 | | | | | | | |
| | M.v. biomass | 0.13 | 1 | 27.06 | 1.28 | kept | 0.2675 | | | | | | | |
| Δ Mineraliz. (0-5cm) | pH | 0.04 | 1 | 20.19 | 0.38 | 1 | 0.5461 | M.v. biomass : Moisture | 0.00 | 1 | 25.30 | 0.01 | 1 | 0.9090 |
| | SOM | 0.13 | 1 | 21.84 | 1.38 | 2 | 0.2522 | M.v. biomass : Nitrate | 0.00 | 1 | 26.04 | 0.03 | 2 | 0.8636 |
| | Number of trees | 0.13 | 1 | 22.04 | 1.35 | 3 | 0.2569 | Nitrate | 1.05 | 1 | 27.95 | 9.79 | kept | 0.0041 |
| | UnderstoryBiom | 0.02 | 1 | 23.32 | 0.25 | 4 | 0.6194 | Moisture | 1.70 | 1 | 27.75 | 15.85 | kept | 0.0004 |
| | LitterBiom | 0.19 | 1 | 24.39 | 2.03 | 5 | 0.1669 | M.v. biomass | 0.00 | 1 | 27.01 | 0.03 | kept | 0.8567 |
| | TreeBA | 0.22 | 1 | 25.04 | 2.33 | 6 | 0.1396 | | | | | | | |
| | Ammonium | 0.31 | 1 | 26.47 | 3.11 | 7 | 0.0891 | | | | | | | |
| | Nitrate | 1.05 | 1 | 27.95 | 9.79 | kept | 0.0041 | | | | | | | |
| | Moisture | 1.70 | 1 | 27.75 | 15.85 | kept | 0.0004 | | | | | | | |
| | M.v. biomass | 0.00 | 1 | 27.01 | 0.03 | kept | 0.8567 | | | | | | | |
| ΔAmmonif. (5-15cm) | LitterBiom | 0.00 | 1 | 20.00 | 0.02 | 1 | 0.9025 | M.v. biomass : TreeBA | 0.00 | 1 | 24.00 | 0.20 | 1 | 0.6598 |
| | Nitrif | 0.01 | 1 | 21.00 | 0.26 | 2 | 0.6188 | M.v. biomass : UnderstoryBiom | 0.01 | 1 | 25.00 | 0.81 | 2 | 0.3777 |
| | Moisture | 0.01 | 1 | 22.00 | 0.42 | 3 | 0.5232 | UnderstoryBiom | 0.34 | 1 | 26.00 | 21.65 | kept | 0.0001 |
| | Ammonium | 0.01 | 1 | 23.00 | 0.46 | 4 | 0.5034 | Nitrate | 0.38 | 1 | 26.00 | 24.25 | kept | 0.0000 |

| | | | | | | | | | | | | | | |
|---|---|---|---|---|---|---|---|---|---|---|---|---|---|---|
| | pH | 0.01 | 1 | 24.00 | 0.64 | 5 | 0.4321 | TreeBA | 0.20 | 1 | 26.00 | 13.15 | kept | 0.0012 |
| | SOM | 0.02 | 1 | 25.00 | 1.06 | 6 | 0.3125 | M.v. biomass | 0.08 | 1 | 26.00 | 5.46 | kept | 0.0274 |
| | Number of trees | 0.02 | 1 | 26.00 | 1.20 | 7 | 0.2833 | M.v. biomass : Nitrate | 0.16 | 1 | 26.00 | 10.10 | kept | 0.0038 |
| | TreeBA | 0.16 | 1 | 27.00 | 7.72 | kept | 0.0098 | | | | | | | |
| | UnderstoryBiom | 0.29 | 1 | 27.00 | 13.96 | kept | 0.0009 | | | | | | | |
| | Nitrate | 0.36 | 1 | 27.00 | 17.54 | kept | 0.0003 | | | | | | | |
| | M.v. biomass | 0.06 | 1 | 27.00 | 3.10 | kept | 0.0896 | | | | | | | |
| Δ Mineraliz. (5-15cm) | Ammonium | 0.00 | 1 | 21.00 | 0.03 | 1 | 0.8580 | M.v. biomass : UnderstoryBiom | 0.00 | 1 | 25.01 | 0.03 | 1 | 0.8623 |
| | Moisture | 0.01 | 1 | 22.00 | 0.16 | 2 | 0.6946 | UnderstoryBiom | 0.47 | 1 | 26.21 | 11.91 | kept | 0.0019 |
| | Number of trees | 0.02 | 1 | 23.00 | 0.41 | 3 | 0.5259 | Nitrate | 0.57 | 1 | 26.29 | 14.49 | kept | 0.0008 |
| | LitterBiom | 0.06 | 1 | 24.00 | 1.53 | 4 | 0.2287 | M.v. biomass | 0.05 | 1 | 26.44 | 1.31 | kept | 0.2634 |
| | SOM | 0.06 | 1 | 24.29 | 1.51 | 5 | 0.2307 | M.v. biomass : Nitrate | 0.23 | 1 | 26.00 | 5.73 | kept | 0.0242 |
| | pH | 0.12 | 1 | 23.04 | 2.82 | 6 | 0.1066 | | | | | | | |
| | TreeBA | 0.14 | 1 | 26.20 | 3.16 | 7 | 0.0872 | | | | | | | |
| | UnderstoryBiom | 0.43 | 1 | 27.26 | 9.30 | kept | 0.0051 | | | | | | | |
| | Nitrate | 0.56 | 1 | 26.17 | 12.05 | kept | 0.0018 | | | | | | | |
| | M.v. biomass | 0.04 | 1 | 27.55 | 0.81 | kept | 0.3760 | | | | | | | |

**Table S4.** Analysis of variance summary from model selection to explain *M.v.* biomass using reference variables. Year is a random effect. Fixed effect coefficients that significantly differ from zero are bold (alpha=.05).

| Resp. var. | Term | SS | Num DF | Den DF | F value | Elim. | p value |
|---|---|---|---|---|---|---|---|
| *M.v.* biomass | Nitrate | 0.22 | 1 | 21.00 | 0.33 | 1 | 0.5698 |
| | Moisture | 0.20 | 1 | 22.00 | 0.32 | 2 | 0.5780 |
| | Ammonium | 0.99 | 1 | 23.00 | 1.59 | 3 | 0.2206 |
| | pH | 0.77 | 1 | 24.00 | 1.20 | 4 | 0.2834 |
| | Number of trees | 1.05 | 1 | 24.09 | 1.68 | 5 | 0.2077 |
| | UnderstoryBiom | 1.79 | 1 | 25.08 | 2.80 | 6 | 0.1064 |
| | LitterBiom | 0.77 | 1 | 25.23 | 1.11 | 7 | 0.3011 |
| | SOM | 1.16 | 1 | 28.00 | 1.67 | 8 | 0.2066 |
| | **TreeBA** | **4.47** | **1** | **29.00** | **6.28** | **kept** | **0.0181** |
| | **Light avail.** | **9.25** | **1** | **29.00** | **12.99** | **kept** | **0.0012** |

**Figure S1.** Each study site consisted of a reference and *M.v.*-invaded plot that were positioned to sample a six-meter transect across the invasion boundary. During peak growing season, August in 2012 and 2013, the three 0.25 m x 0.25 m quadrats were aligned 1 m apart and perpendicular to the invasion boundary. From each quadrat, M.v. and non-M.v. understory vegetation were harvested, plant litter was collected, and soil was sampled. In 2012, trees at each site were surveyed in within a 10 meter radius centered on the 2012 transect invasion boundary. In 2012, light availability was measured at 1m above each quadrat and averaged by plot.

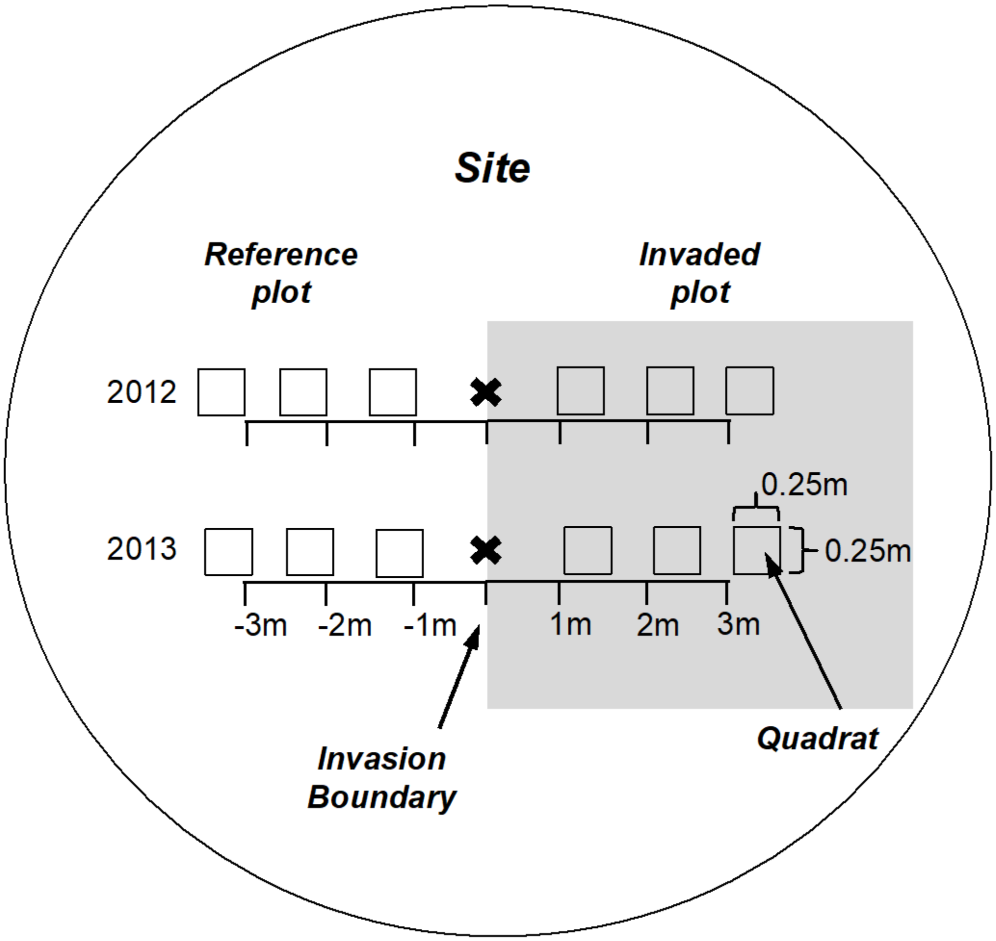

**Figure S2.** Aggregated across study sites, panels show variation, or lack thereof, in soil inorganic N pools and fluxes in reference and invaded plots by year (2012 = circle and solid line, 2013 = triangle and dashed line) and soil depth (0-5cm = black, 5-15cm = gray). Invaded plots tend to have higher ammonification rates (0-5cm) and marginally higher mineralization rates (5-15cm) (Table S1); mean ± SE.

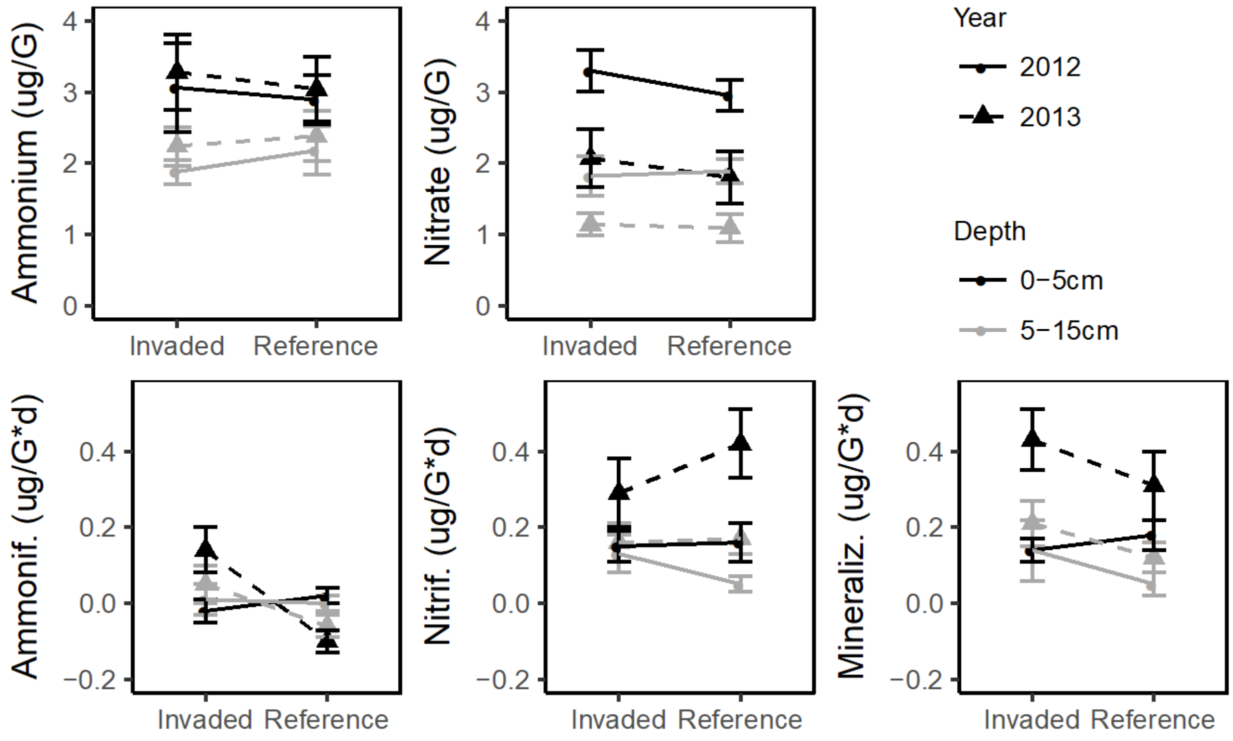

**Figure S3.** Piecewise SEMs constructed for Δ nitrification (0-5cm) and Δ ammonification (5-15cm) using regressions 1 & 2. The data adequately fit each model (C = 7.23, 10.95; df = 8, 6; p > .05). Values on top of arrows indicate the standardized path coefficient and solid bold arrows are significant (alpha = .05); marginal $R^2$ values are provided. An arrow that points to another arrow illustrates an interaction term (e.g. Fig. 1, arrow D). Tree BA is shorthand for "Tree basal area". Path diagrams built to understand Δ nitrification (0-5cm) and Δ ammonification (5-15cm) are analogous to the mineralization results presented in Fig. 2. The significant paths between nitrate and Δ nitrification (0-5cm) (panel a) should be considered with caution since the highest nitrate and moisture sample is very influential (see Fig. S5).

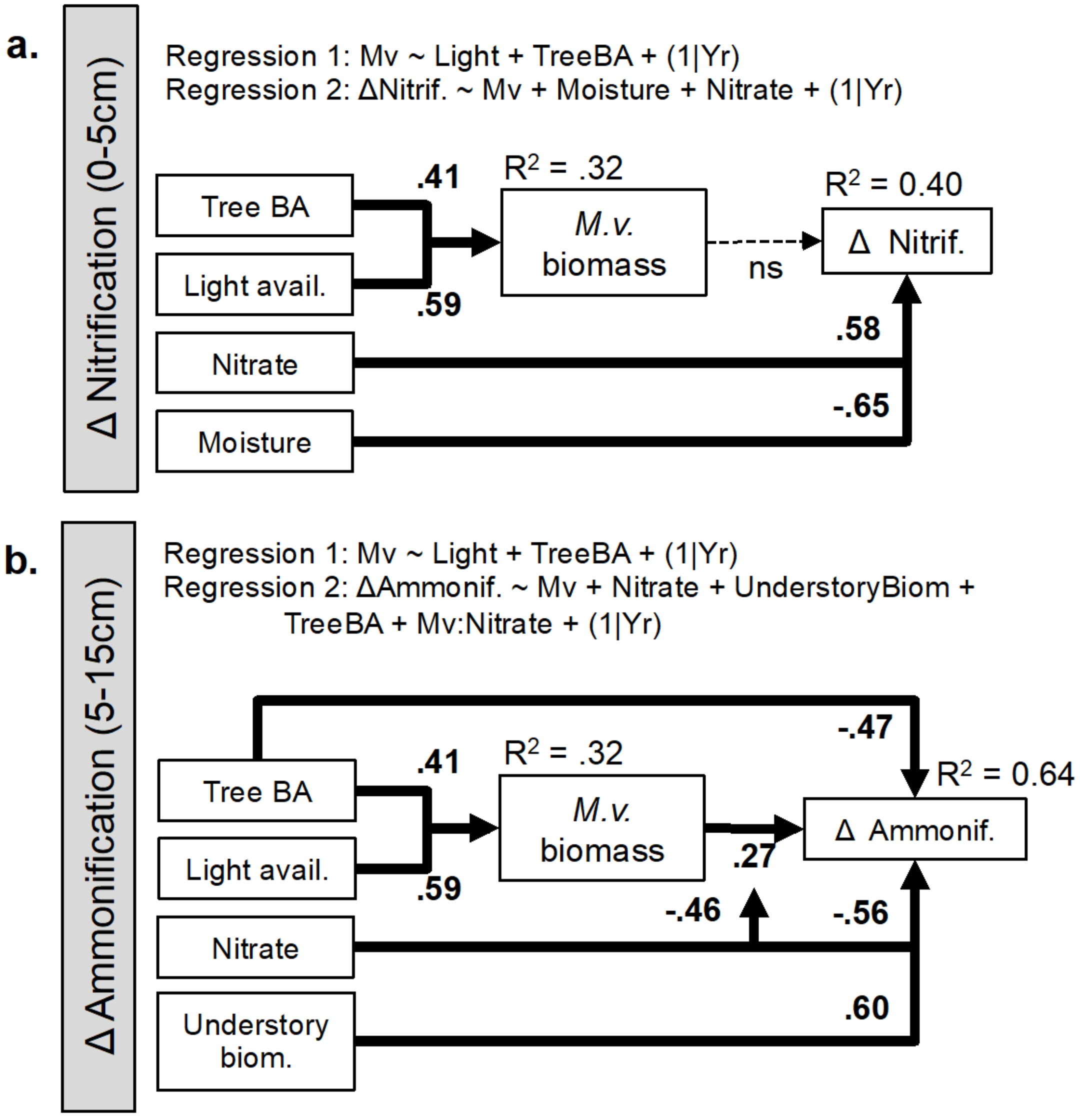

**Figure S4.** Initial piecewise SEM constructed for Δ mineralization (5-15cm). Regression 2 is based on the final model proposed by model selection procedures (Table S3). The data do not adequately fit the SEM model (C = 18.34; df = 8; p = .02). For this reason, a causal link was subsequently added between TreeBA and Δ mineralization (Fig. 2; C = 10.22; df = 6; p > .05). Values on top of arrows indicate the standardized path coefficient and solid bold arrows are significant (alpha = .05); marginal $R^2$ values are provided. An arrow that points to another arrow illustrates an interaction term (e.g. Fig. 1, arrow D). Tree BA is shorthand for "Tree basal area".

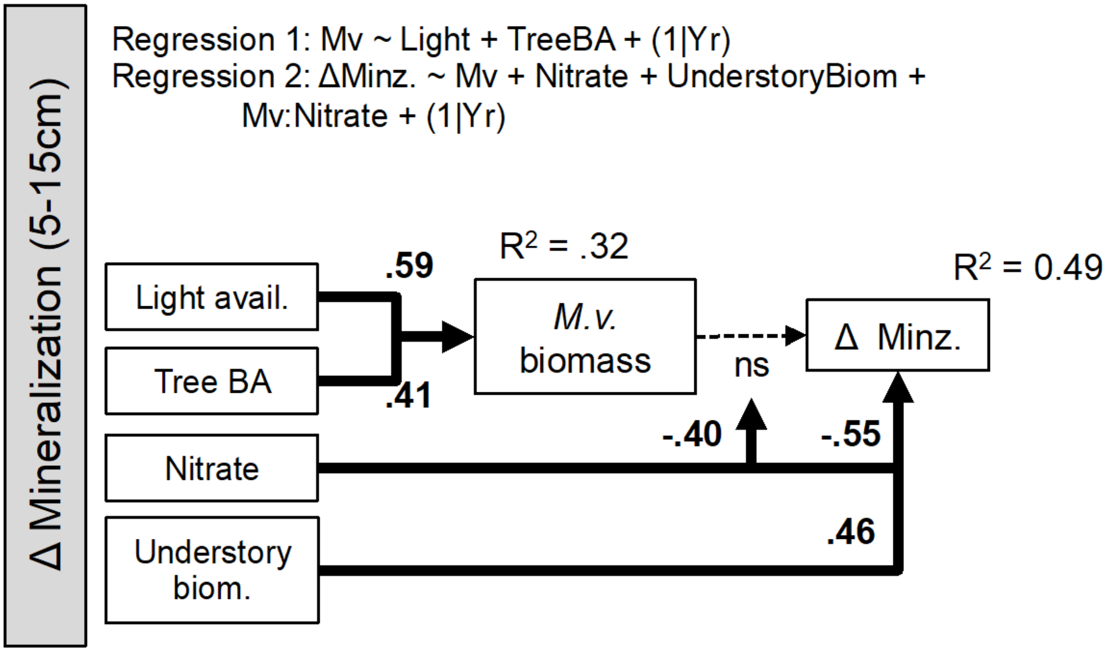

**Figure S5.** (a, b) Nitrification (0-5cm) and (c, d) mineralization (0-5cm) differences between paired reference and invaded plots are best explained by reference soil (a, c) nitrate and (b, d) moisture (Table S3). Each point represents a site (n = 16) and year (2012 = circles, 2013 = triangles). Model fits and 95% prediction intervals are shown conditional on year (2012 = solid line, 2013 = dotted line; prediction intervals overlap in this figure). Significant relationships presented in panels a, c, and d should be considered with caution since the highest nitrate and moisture sample is very influential.

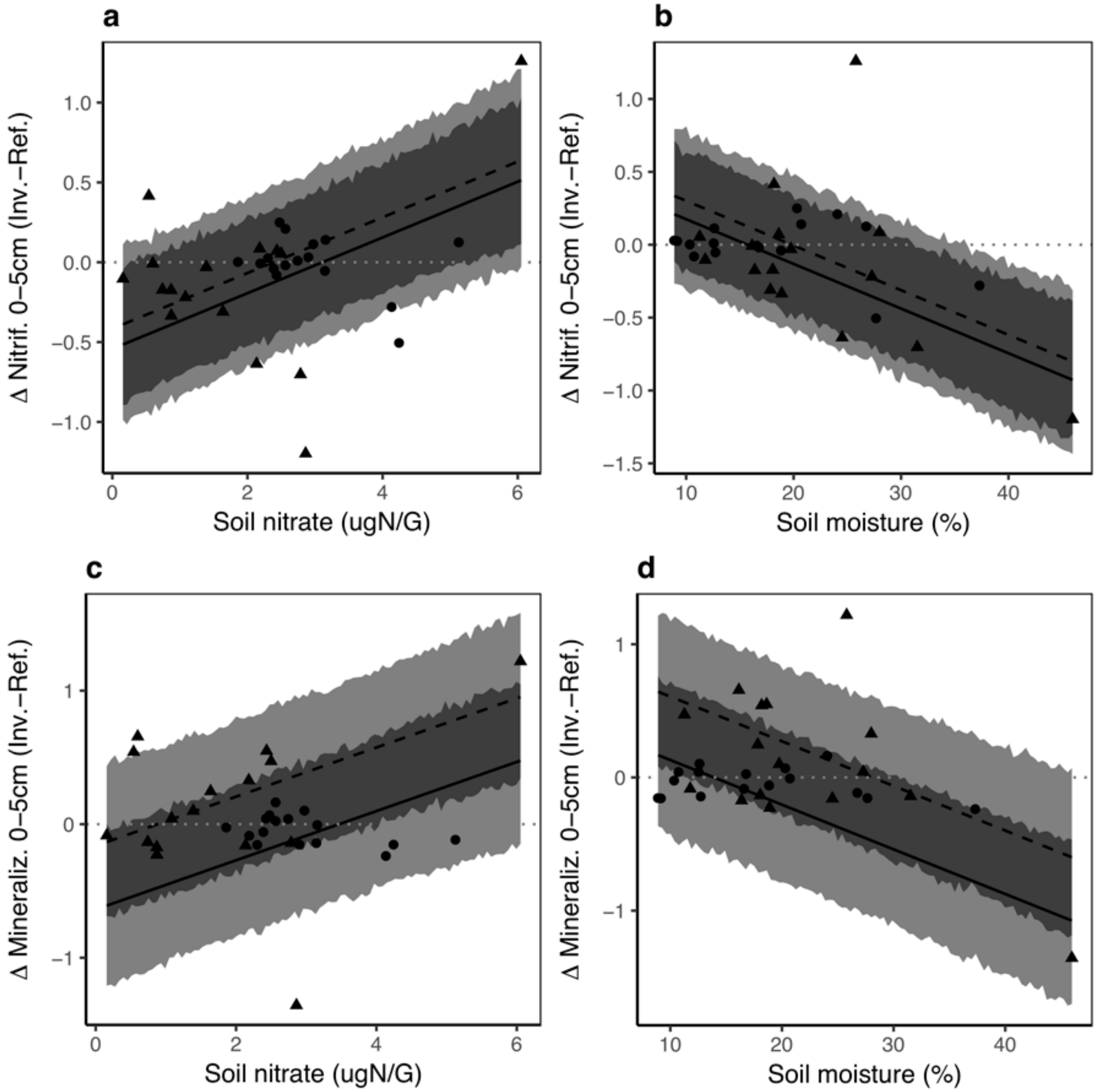

**Figure S6.** Ammonification (5-15cm) differences between paired reference and invaded plots are best explained by (a) an interaction between *M.v.* biomass and reference soil nitrate, (b) tree basal area, and (c) understory biomass (Table S3). Patterns associated with Δ mineralization at 5-15cm are analogous (Fig. 3). Each point represents a site (n = 16) and year. For panel a, lighter point color represents larger soil nitrate concentrations. For panels b and c, model fits and 95% prediction intervals are shown conditional on year (2012 = solid line, 2013 = dotted line; prediction intervals overlap in this figure).

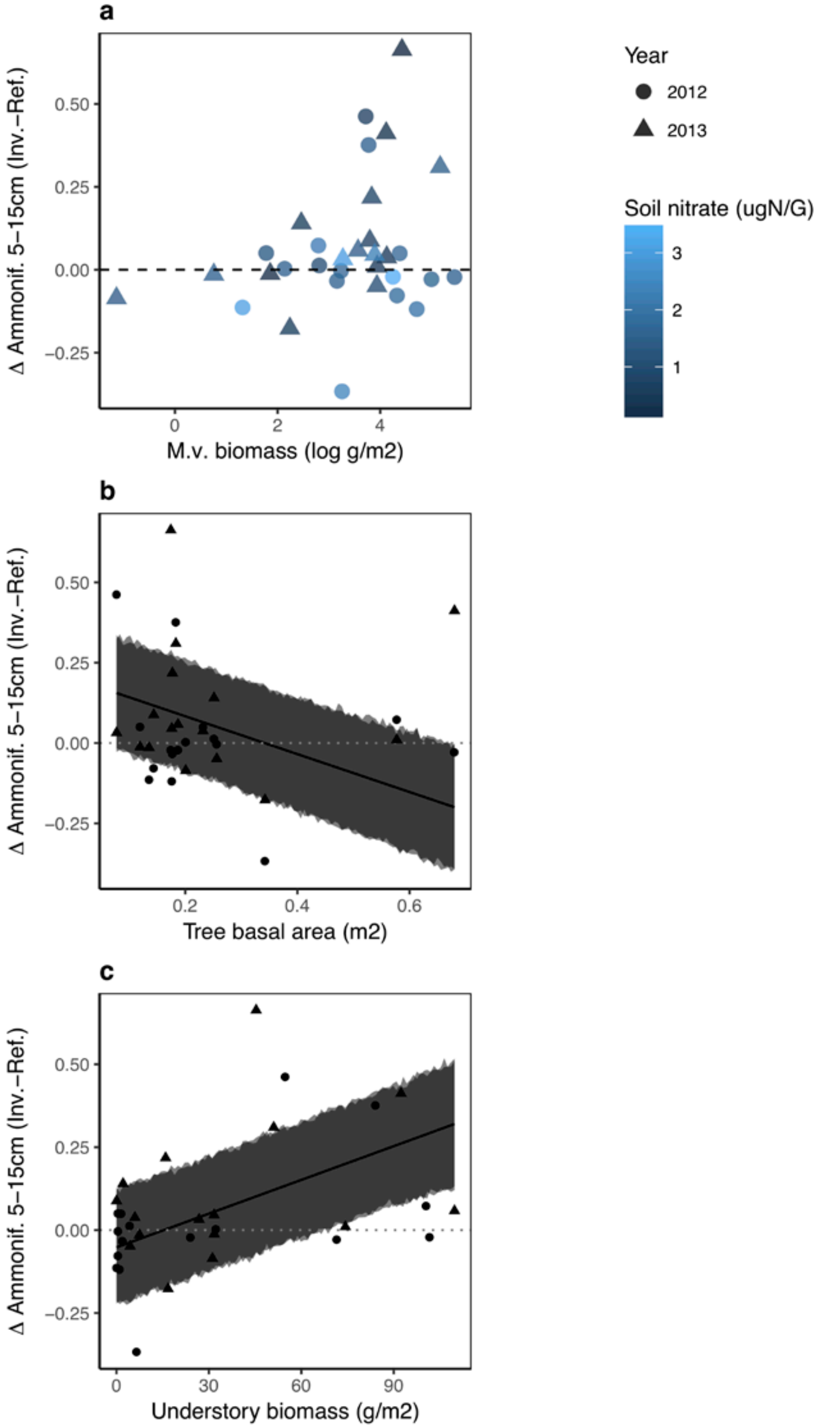

**Figure S7.** *M.v.* biomass is best explained by light availability (%) and tree basal area (m2) (see Fig 3d). *M.v.* biomass increases with more light availability ($p < 0.05$). Each point represents a site (n = 16) and year (2012 = circles, 2013 = triangles). Model fits and 95% prediction intervals are shown, conditional on year (2012 = solid line, 2013 = dotted line; prediction intervals overlap in this figure).

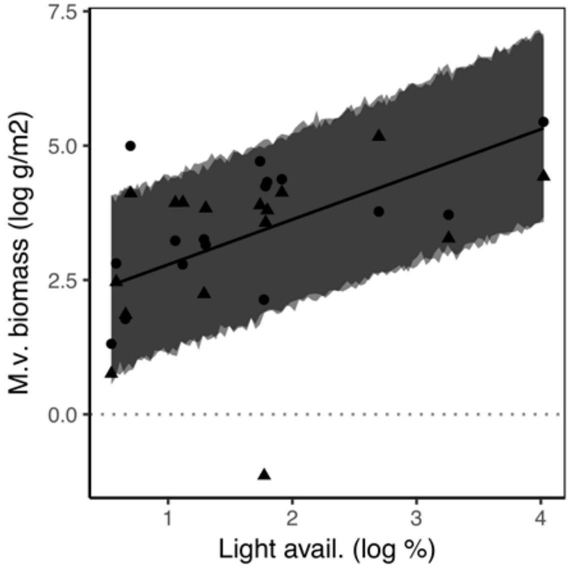

**Figure S8.** Relationship between tree basal area (m2), light availability (log %), and understory biomass (g/m2). For panels a, each point is a site (n = 16). For panels b-c, each point is a site and year (2012 = circles, 2013 = triangles) with gray lines connecting samples from the same site. Understory biomass increases with tree basal area (panel b, p = 0.01). All other relationships are non-significant.

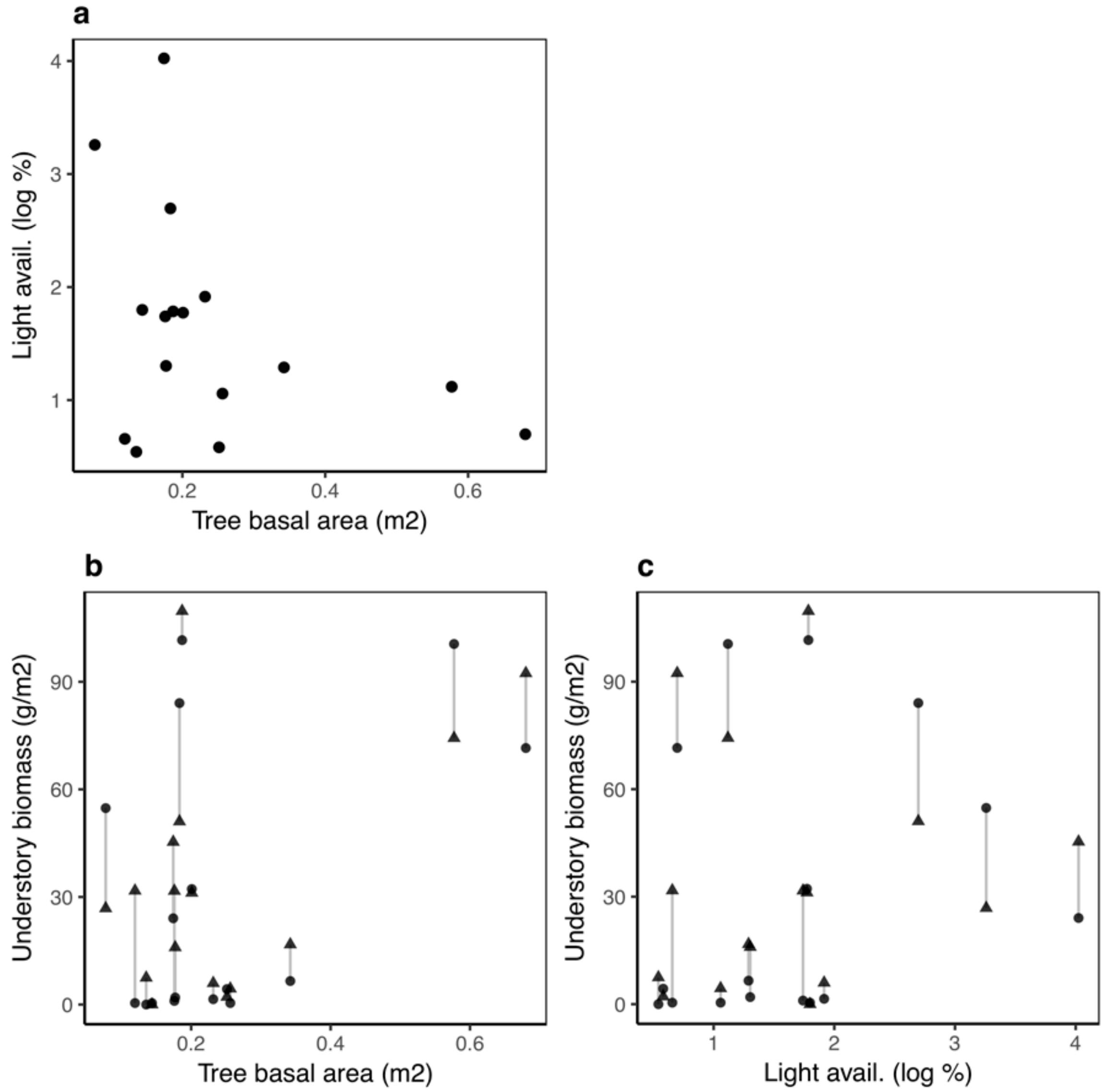

**Figure S9.** Relationship between understory biomass (g/m2) and invader biomass (g/m2) across sites has a weak hump-shape. Each point is a site (n = 16) and year (2012 = circles, 2013 = triangles) with gray lines connecting samples from the same site.

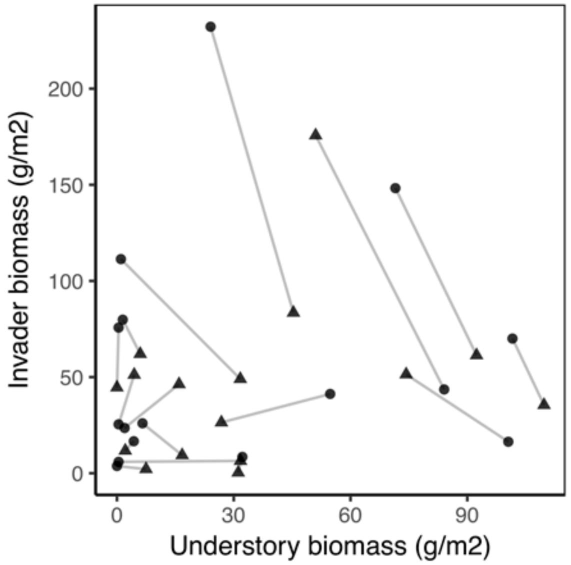

Supplement: supplement [file NIHMS1944183-supplement-supplement.pdf]
